# Biomimickry of UPEC Cytoinvasion: A Novel Concept for Improved Drug Delivery in UTI

**DOI:** 10.3390/pathogens5010016

**Published:** 2016-02-04

**Authors:** Clara Maria Pichl, Bernhard Dunkl, Bernhard Brauner, Franz Gabor, Michael Wirth, Lukas Neutsch

**Affiliations:** Department of Pharmaceutical Technology and Biopharmaceutics, University of Vienna, Althanstr. 14, 1090 Vienna, Austria; bernhard.dunkl@aon.at (B.D.); bernhard.brauner@gmx.at (B.B.); franz.gabor@univie.ac.at (F.G.); michael.wirth@univie.ac.at (M.W.); lukas.neutsch@tuwien.ac.at (L.N.)

**Keywords:** urinary tract infection, UPEC, lectin, instillation, drug delivery, WGA

## Abstract

Urinary tract infections (UTIs) are among the most common bacterial infections. In an increasing number of cases, pathogen (multi-)resistance hampers durable treatment success via the standard therapies. On the functional level, the activity of urinary excreted antibiotics is compromized by the efficient tissue colonization mechanism of uropathogenic *Escherichia coli* (UPEC). Advanced drug delivery systems aim at exploiting a glycan-mediated targeting mechanism, similar to the UPEC invasion pathway, to increase bioavailability. This may be realized by conjugation of intravesically applied drugs or drug carriers to chosen plant lectins. Higher local drug concentrations in or nearby bacterial reservoirs may be gained, with higher chances for complete eradication. In this study, preliminary parameters to clarify the potential of this biorecognitive approach were evaluated. Glycan-triggered interaction cascades and uptake processes of several plant lectins with distinct carbohydrate specificities were characterized, and wheat germ agglutinin (WGA) could be identified as the most promising targeter for crossing the urothelial membrane barrier. In partially differentiated primary cells, intracellular accumulation sites were largely identical for GlcNAc- and Mannose-specific lectins. This indicates that WGA-mediated delivery may also enter host cells via the FimH-dependent uptake pathway.

## 1. Introduction

Urinary tract infections (UTIs) rank among the most common bacterial infections and represent a severe burden to the society, with significant impact on patient quality of life and overall health care costs [1]. Despite a wide range of established treatment schedules, problems with antibiotic multiresistance are increasing. Functionally, the difficulty lies in the struggle to achieve complete tissue eradication. In UTI, a key step in host colonization by uropathogenic *Escherichia coli* (UPEC) is the mannose-mediated adhesion of the bacterial FimH lectin domains to membrane proteins and lipids. This FimH/carbohydrate interaction can trigger active uptake processes, allowing the bacteria to cross the rigid membrane barrier and proliferate in cytosolic or membrane-bound compartments, where they are protected from antibiotics and the host immune system. Release from these reservoirs back into the bladder lumen after a certain lag time is considered a main cause for recurrent infections [2,3].

**Figure 1:**
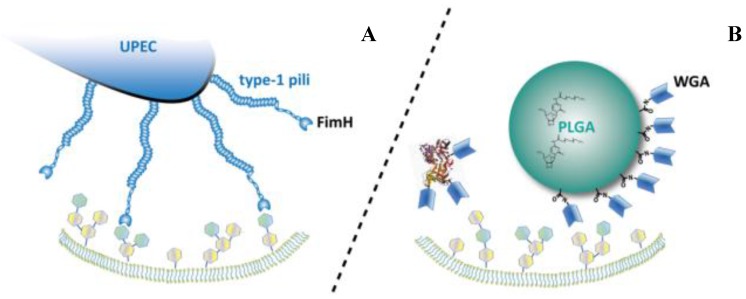
The basic principle of biomimetic delivery systems via carbohydrate targeting. Uropathogenic *Escherichia coli* (UPEC) cytoadhesion mediated by the natural FimH-lectin-domain (**A**). Binding of lectin-functionalized drug carriers or proteins to the cellular surface (**B**). (PLGA: *Poly(lactic-co-glycolic acid)*; WGA: *Wheat germ agglutinin*).

The increasing prevalence of problematic cases calls for the development of novel therapeutic strategies that allow for a more efficient delivery of the antibiotic drug to the bacterial reservoirs. Today, intravesical instillations of antibiotics are mainly adopted as salvage therapy in complex (recurrent/multiresistant) cases, and offer the advantage of providing direct access to the diseased tissue [4]. However, drugbioavailability is compromized by constant washout and dilution with freshly secreted urine. The use of delivery systems that can prolong the local residence time and mediate a regional focusing on the UPEC accumulation sites may offer interesting perspectives to overcome these drawbacks. For maximum activity, a delivery system should closely follow the uptake routes of the bacterial pathogen. By mimicking the function of FimH, which is the key adhesin in bacterial trafficking, this concept may soon become reality. The mechanism of the FimH protein may be imitated by using plant-derived carbohydrate-binding proteins, so called lectins. The specific interaction between lectins and cellular carbohydrates might confer cytoinvasive properties to the conjugated antibiotics or microparticulate drug carriers, which facilitates uptake into intracellular compartments (Figure 1).

In this study, we aimed to elucidate the potential of this biorecognitive approach in a well-defined and easy-to-monitor tissue culture model. Due to broad availability and a (relatively) close analogy to human systems [5], primary porcine urothelial cells were chosen as the basis for preliminary investigations. In initial screening studies, the binding behaviour of several plant lectins with distinct carbohydrate specificity was characterized. A particular focus was set on internalisation studies to evaluate in higher detail the mechanisms involved in uptake and intracellular trafficking of the selected lectins. Colocalization assays were conducted to assess potential similarities and differences in the delivery pathways of the different lectins.

## 2. Results and Discussion

In screening assays, WGA was identified as the most promising targeter due to extensive and stable cell binding. The glycan-mediated targeting approach was shown to bear advantages as compared to non-specific protein/cell interactions, e.g., observed for albumin. Overall, the experimentally determined lectin binding capacity decreased in order: WGA >> LCA > UEA > PNA > GNL > DBA (LCA: *Lens culinaris agglutinin*; UEA: *Ulex europaeus agglutinin*; PNA: *Peanut agglutinin*; GNL: *Galanthus nivalis lectin*; DBA: *Dolichus biflorus agglutinin*). Competitive inhibition studies with complementary carbohydrates were carried out to confirm the specificity of the binding process and gain information on the average binding affinity.

**Figure 2:**
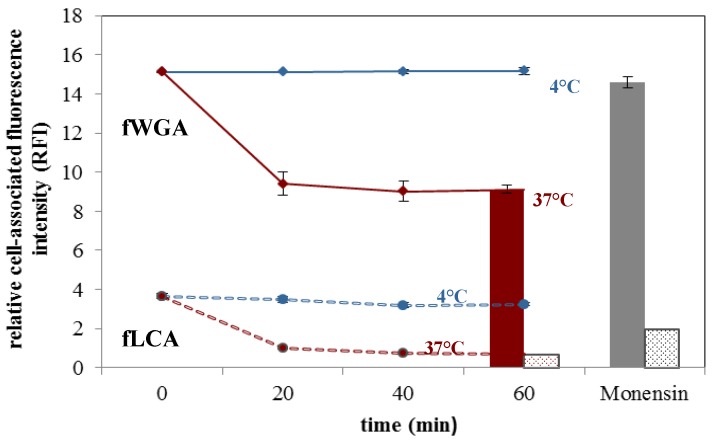
Cellular internalization of Fluorescein-labelled *Wheat germ agglutinin* (fWGA) (solid lines and solid bars) and Fluorescein-labelled *Lens culinaris* (fLCA) (dashed lines and hatched bars). Lectins exhibit durable binding at 4 °C (fWGA: solid blue line, fLCA: dashed blue line; *0 to 60 min*), whereas a decrease in fluorescence intensity can be observed at 37 °C (fWGA: solid red line, fLCA: dashed red line; *0 to 60 min*). This decrease is largely mediated by acidic quenching ,which can be restored by addition of the ionophor monensin (red bars: *quenched state at 60 min*; grey bars: *quenching restored by Monensin*) (mean ± SD, n = 3). Data of single cells, assessed via flow cytometry.

Fluorescence-based internalization assays were performed to examine up to which extent a given lectin targeter is internalized after adhering to the urothelial cell surface. In the present study, the uptake behaviour of fluorescein-labelled WGA (fWGA) and LCA (fLCA) were compared in detail, since WGA harbours the highest binding potential (mainly via GlcNAc) and LCA targets glycan epitopes similar to those recognized by FimH (mannose) [6]. Quenching ratios around 40% for fWGA and 71% for fLCA pointed to an efficient uptake into late endosomal/lysosomal compartments. However, a total recovery of the quenched signal by ionophor treatment could not be achieved, indicating that also other uptake routes may contribute to the overall effect (Figure 2). The uptake of the lectins was additionally confirmed via fluorescence microscopy.

**Figure 3:**
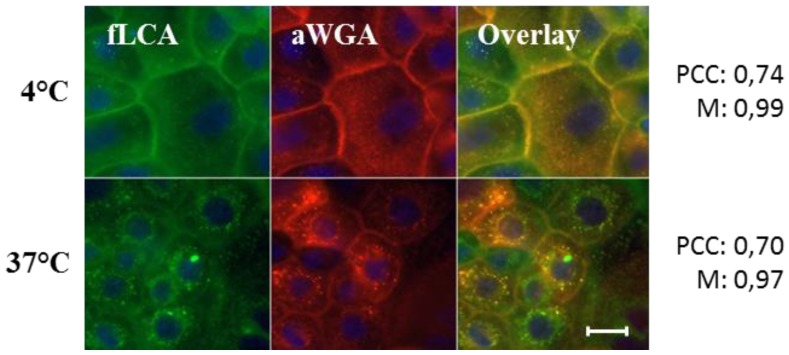
Colocalization analysis of fLCA and aWGA after binding (4 °C) and internalization (37 °C) on primary porcine urothelial cell monolayers. Colocalization was evaluated on basis of the Pearson’s correlation coefficient (PCC) and Manders’colocalization coefficient (M). Scale bar represents 20 µm.

Due to its 4-fold higher binding capacity in comparison to LCA (cf. Figure 2) and its promising uptake behaviour, WGA may be particularly suited as a targeter for novel drug delivery systems. However, since the specific binding of WGA to N-acetyl-D-glucosamine and sialic acid residues may be functionally different from the mannose-specific binding of UPEC, further research on the comparison of the trafficking routes of WGA and LCA is warranted. Co-incubation of an Alexa Fluor^®^ 594/647 conjugate of WGA (aWGA) and fLCA resulted in a near-complete overlap of the individual channels, indicating a high parallelism in the processing pathway of both lectins. In a metabolically quiescent state (4 °C), mere staining of the cell surface was visible, whereas at 37 °C intracellular clustering pointed to endocytotic uptake (Figure 3).

These results confirm that, despite being directed to different carbohydrate epitopes, WGA may ultimately reach the same compartments or accumulation sites as are reached via the mannose-mediated uptake of FimH-piliated bacteria. Furthermore, the robust nature, conformational stability and availability of multiple binding pockets in the protein renders WGA particularly suited for use in coupling chemistry and carrier modification.

Successful surface-functionalization with WGA and enhanced binding rates of the prepared microcarriers could be verified in preliminary results (studies ongoing, unpublished data). The suggested administration of the prepared delivery vehicles via intravesical instillation facilitates a localized administration to the site of disease, exposing pathogens to high concentrations of the active agent. Hence, the use of lectins for biorecognitive targeting strategies might indeed increase the chances for good response. The stable bioadhesion of the applied vehicle might not only counteract the harsh washout conditions in the urinary tract, but may also facilitate a continuous release of the drug and hence lead to a better concentration-over-time profile.

Moreover, a direct lectin/API conjugation can be realized, as demonstrated in other areas of application [7]. Internalization of lectins has been observed in various other tissues, including the brain and intestine [8,9]; however, considering the barrier-function of the urothelium, a systemic distribution starting from the bladder lumen is highly improbable, which minimizes the risk of associated side effects.

WGA-decorated, biodegradable nano- or microparticulate drug carriers may, in addition to facilitating stable binding, allow fragile compounds to withstand the hostile environmental conditions in the urinary tract. The use of such advanced delivery systems could greatly increase therapeutic outcome in the treatment of UTIs.

## 3. Experimental Section

### 3.1. Chemicals

Fluorescein-labelled *Wheat germ agglutinin* (fWGA) and *Lens culinaris agglutinin* (fLCA) were purchased from Vector Laboratories (Burlingame, CA, USA). All other chemicals were obtained from Life Technologies (Carlsbad, CA, USA) or Sigma (St. Louis, MO, USA). Porcine bladders obtained from a local abattoir and were processed according to an established protocol [10] with slight modifications to isolate urothelial cells.

### 3.2. Glycan-Mediated Uptake of Cell-Bound Lectins

The energy-dependent internalization of fWGA and fLCA was studied by assessing the acidic fluorescein quench upon uptake in intracellular compartments. Single cells were incubated with the respective lectin (60 nM per 2.5 × 10^5^ cells, 50 µL) for 30 min at 4 °C (surface loading). After washing with PBS, cells were either kept at 4 °C or warmed to 37 °C to initiate energy-dependent uptake. Endosomal/lysosomal accumulation was followed via flow cytometry using the restored fluorescence signal (monensin, 20 µM, 50 µL) as a control.

### 3.3. Colocalization Analysis

Colocalization studies with an Alexa Fluor® (Thermo Fisher Scientific, Waltham, MA, USA) 594/647 conjugate of WGA (aWGA) and fLCA were conducted to compare their uptake and intracellular localization. Confluent cell monolayers on glass coverslips were washed and surface-loaded with 50 µL of aWGA/fLCA solution (12.5 nM each) for 30 min at 4 °C. After thorough washing, cells were further incubated for 60 min at 37 °C or 4 °C. Imaging studies were carried out using a deconvolution microscopy system.

## 4. Conclusions

The results of this study indicate that plant lectins may indeed be utilizable as mediators of a FimH-like uptake stimulation. Prolonged residence times and enhanced intracellular urothelial accumulation can be achieved via the specific targeting of surface-accessible carbohydrates. This may ultimately allow for counteracting limitations in existing UTI treatment schedules, such as the loss of drug by urinary excretion. Advanced delivery systems that follow the UPEC invasion pathway may in future constitute a potent strategy to eradicate multiresistant UPEC in their hard-to-reach reservoirs. Currently, surface-functionalized microparticulate carrier systems are being tested for lectin-targeted administration of antibiotics in complex cases of disease.
